# The Need for Physiological Micro-Nanofluidic Systems of the Brain

**DOI:** 10.3389/fbioe.2019.00100

**Published:** 2019-05-07

**Authors:** Jean-Philippe Frimat, Regina Luttge

**Affiliations:** ^1^Neuro-Nanoscale Engineering Group, Microsystems Section & ICMS Institute for Complex Molecular Systems, Eindhoven University of Technology, Eindhoven, Netherlands; ^2^Department of Neurosurgery, Maastricht University Medical Centre, School for Mental Health and Neuroscience, Eindhoven, Netherlands

**Keywords:** brain models, brain-on-a-chip, micro- and nanofluidics, organ-on-a-chip, organoids

## Abstract

In this article, we review brain-on-a-chip models and associated underlying technologies. Micro-nanofluidic systems of the brain can utilize the entire spectrum of organoid technology. Notably, there is an urgent clinical need for a physiologically relevant microfluidic platform that can mimic the brain. Brain diseases affect millions of people worldwide, and this number will grow as the size of elderly population increases, thus making brain disease a serious public health problem. Brain disease modeling typically involves the use of *in vivo* rodent models, which is time consuming, resource intensive, and arguably unethical because many animals are required for a single study. Moreover, rodent models may not accurately predict human diseases, leading to erroneous results, thus rendering animal models poor predictors of human responses to treatment. Various clinical researchers have highlighted this issue, showing that initial physiological descriptions of animal models rarely encompass all the desired human features, including how closely the model captures what is observed in patients. Consequently, such animal models only mimic certain disease aspects, and they are often inadequate for studying how a certain molecule affects various aspects of a disease. Thus, there is a great need for the development of the brain-on-a-chip technology based on which a human brain model can be engineered by assembling cell lines to generate an organ-level model. To produce such a brain-on-a-chip device, selection of appropriate cells lines is critical because brain tissue consists of many different neuronal subtypes, including a plethora of supporting glial cell types. Additionally, cellular network bio-architecture significantly varies throughout different brain regions, forming complex structures and circuitries; this needs to be accounted for in the chip design process. Compartmentalized microenvironments can also be designed within the microphysiological cell culture system to fulfill advanced requirements of a given application. On-chip integration methods have already enabled advances in Parkinson's disease, Alzheimer's disease, and epilepsy modeling, which are discussed herein. In conclusion, for the brain model to be functional, combining engineered microsystems with stem cell (hiPSC) technology is specifically beneficial because hiPSCs can contribute to the complexity of tissue architecture based on their level of differentiation and thereby, biology itself.

## Introduction

We suggest that there is an urgent medical need for physiologically relevant microfluidic platforms that can mimic the brain. Therefore, what are the technical arguments for following such a radically new approach? A previous World Health Organization (WHO) report showed that neurological disorders, ranging from epilepsy, Alzheimer's disease, and stroke to headache, affect up to one billion people worldwide (Dua et al., 2006). An estimated 6.8 million people die every year due to neurological disorders. In 2004, the economic cost of neurological diseases in Europe was estimated at between 139 and 386 billion euros (Andlin-Sobocki et al., 2005).

Research with models of brain disorders typically involves the use of rodents *in vivo*, which is time consuming, resource intensive, and arguably unethical because many animals are required for a single study (Festing and Wilkinson, 2007). Moreover, rodent models may not accurately predict human disease and may lead to erroneous results (i.e., false positives; Perrin, 2014), rendering such animal models as poor predictors of human responses. Various clinical researchers have highlighted this issue, showing that the initial physiological descriptions of animal models rarely encompass all of the desired human features, including how closely the model captures what is observed in patients. Consequently, such animal models are often inadequate for studying how a certain molecule affects various aspects of a disease (Perrin, 2014). Thus, there is presently a great need for the development of better models to investigate the brain and its diseases.

In neurobiological research, microfluidic channels and various interconnected compartment geometries have been used to study axon guidance (Francisco et al., 2007) and neuronal regeneration processes (Taylor et al., 2003, 2005). Spatiotemporal investigations of electrophysiological function have also been performed on microelectrode arrays (MEAs) (Ban et al., 2007; van Vliet et al., 2007). We recently introduced organ-on-a-chip technology, which yields miniaturized systems that support two- and three-dimensional (2D and 3D) cell culture formats (Frimat et al., 2015; Bastiaens et al., 2018; Moonen et al., 2018; Xie et al., 2018). These on-chip low-volume culture systems can forward-engineer brain-like tissues as well as other organ features from a small number of human cells and simply rely on internal diffusion facilitated by the microfluidic approach (Ronaldson-Bouchard and Vunjak-Novakovic, 2018). A lung-on-a-chip study demonstrated the use of stem cells assembled to provide an organotypic model resembling full organ structure rather than only mimicking certain aspects of organ function (Huh et al., 2010). Although this study is a fascinating development of our era, it had a basic science scope concerning disease modeling. Micro- and nanotechnologies have significantly contributed to the development of better human organ and disease models. However, to successfully engineer a brain-on-a-chip model, researchers must produce an *in vitro* model that accurately mimics critical cellular events observed *in vivo*. Therefore, to construct functional brain tissue within a miniaturized system, certain criteria must be met.

Considering that brain disorders are the number one factor reducing quality of life in aging societies, we review the advances in and requirements of microsystems that mimic brain function. First, we summarize the literature concerning the essential technical features involved in the design of brain-on-a-chip systems. Second, we address the clinical requirements of and medical need for brain-on-a-chip systems by reviewing previous applied studies. Finally, we argue that there is not only a clear need for brain-on-a-chip technology in biomedical research, but also, given the dedicated efforts of engineers to improve the performance of brain-on-a-chip devices as well as their high biological and clinical relevance, technological solutions can be achieved.

## Brain-on-a-Chip Technology and Brain Models

As previously mentioned, a brain-on-a-chip is a micro-engineered chip platform that mimics the physiological microenvironment and tissue of a particular brain region. In this section, we discuss detailed brain-on-a-chip design features and culture methods, including their applications to brain disease modeling.

### Conventional Methods for 3D Neuronal Cell Cultures

**Figure 1:**
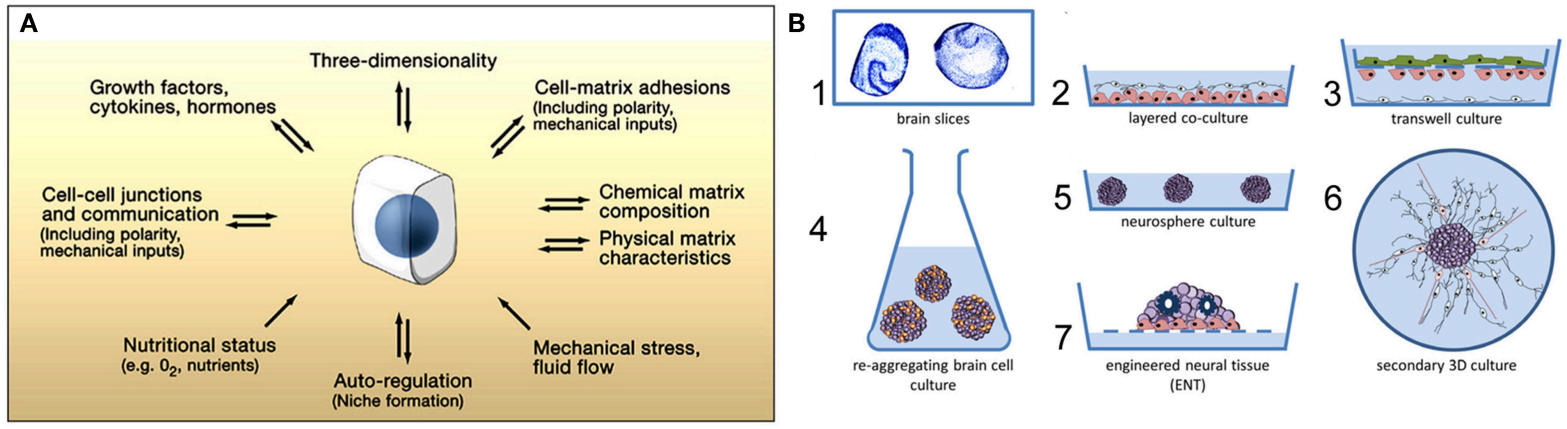
**(A)** Microenvironmental factors affecting cell behavior. Numerous spatially and temporally changing microenvironmental aspects may affect how accurately a 3D model reflects cellular behavior *in vivo*. Conversely, cells (center) can actively modify their local microenvironment. Figure reprinted with permission, previously published in Cell, Yamada and Cukierman (2007). **(B)** Conventional methods for designing 3D *in vitro* models of the nervous system. The first 3D *in vitro* models used rodent brain slices (1) or co-culture models consisting of neurons placed directly on top of a glial cell layer (2). Transwell culture models are also used to mimic the blood-brain barrier *in vitro* (3). Re-aggregating brain cell culture models can also be formed by the spontaneous re-aggregation of dissociated rodent brain cells (4). Neurospheres can be generated and kept in low-adherence plastic plates (5) with secondary 3D cultures being produced by plating these neurospheres onto a planar adhesive substrate (6). Engineered neural tissue is generated by growing highly concentrated stem cell suspensions on a membrane floating at the air-liquid interface. This tissue is polarized (e.g., astrocytes at the bottom), consists of several neuronal subtypes, and shows rosettes as neural tube-like structures (7). Figure reprinted with permission, previously published in ALTEX, Alépée et al. (2014).

When designing brain models, cellular mass transport (Yamada and Cukierman, 2007) is an essential aspect to consider to engineer the correct microenvironment for different cellular events (Figure 1A). Various technologies exist that mainly attempt to mimic the *in vivo* microenvironment of the central nervous system (CNS). Alépée et al. (2014) reviewed the conventional methods for designing organotypic brain models (Figure 1B). For example, electrophysiological recordings of neuronal tissues primarily rely on rodent brain slices (Qi et al., 2019). Co-culture models with a glial cell layer overlaid by a second neuron layer have also been studied (Viviani, 2006). To model the blood-brain barrier, transwell culture systems have been developed in which neurons and endothelial cells separated by a porous membrane can be grown and permeability assays as well as transendothelial electrical resistance (TEER) measurements can be performed (Patabendige et al., 2013). Dissociated rodent brain cells have also been used with the development of methods to isolate and re-aggregate 3D brain cell cultures (Bart Schurink and Luttge, 2013). Moreover, neurospheres can be grown on low-adherence plastic plates and then replated onto an adhesive substrate, which supports the outgrowth of radial glia and migrating neurons (Jensen and Parmar, 2006). Finally, using stem cell technology, neuronal tissue can spontaneously self-assemble into organoids (Lancaster et al., 2013), which will be discussed in section Human Induced Pluripotent Stem Cell Technology. However, these methods are insufficient and too reductionist for disease modeling.

### Chip Technologies Using Various Combination of Microfluidics, Electrode Arrays, and 3D Cell Cultures

**Figure 2:**
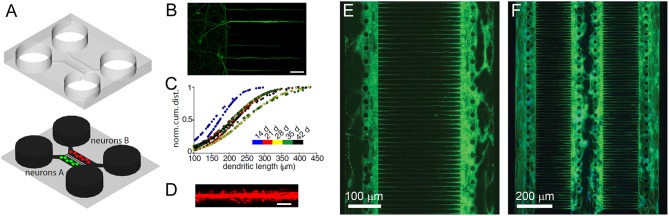
Dendrites grow into the microgrooves of microfluidic chambers. **(A)** Schematic of a microfluidic chamber. **(B)** Fluorescence image of dendrites extending into microgrooves (MAP2, green; scale bar, 50 μm). **(C)** Dendritic length within microgrooves as a function of days in culture. **(D)** Fluorescence image of dendritic spines within microgrooves (scale bar, 10 μm). Figure reprinted with permission, previously published in Neuron, Taylor et al. (2010). Highly interconnected SH-SY5Y co-cultures in two **(E)** and three **(F)** compartments. Neurons and neurite outgrowths are immunostained for β-III tubulin following culture for 5 days. Figure reprinted with permission, previously published in Lab on a Chip, Dinh et al. (2013).

To a large extent, brain model advances have been limited due to a lack of controlled environments which recreate CNS microenvironment characteristics. The established cell culture models mimicking brain function are too simplistic, whereas more physiologically relevant approaches, such as the use of *ex vivo* brain slices or *in vivo* experiments, provide limited control and make information extraction difficult. Therefore, advances in nano- and microfabrication technology have increased the developmental potential of brain-on-a-chip devices (Figures 2A–D; Park et al., 2006). These advances include microfluidic platforms that have been engineered for different neuroscience research needs, such as greater visualization (Lu et al., 2012) and quantification (Park et al., 2014; Zhao et al., 2014), network formation control (Frimat et al., 2010; Dinh et al., 2013), studying neuronal co-culture effects (Figures 2E,F; Dinh et al., 2013), improving brain slice culture performance, and examining on-chip electrophysiology (Massobrio et al., 2016). The development of compartmental culturing platform for primary neurons, which combines microfluidics with surface patterning (Figure 2), has allowed for real-time monitoring of axons (Zhao et al., 2014) and synapses (Taylor et al., 2010) as well as studies of brain injury and trauma (Taylor et al., 2005). Another important platform is the transwell assay, which enables the study of organ membrane function (Jang et al., 2013). The development of microfabrication methods yielding novel platforms for complex tissue constructs, such as the blood brain barrier (BBB), has received considerable attention (Banks, 2016).

**Figure 3:**
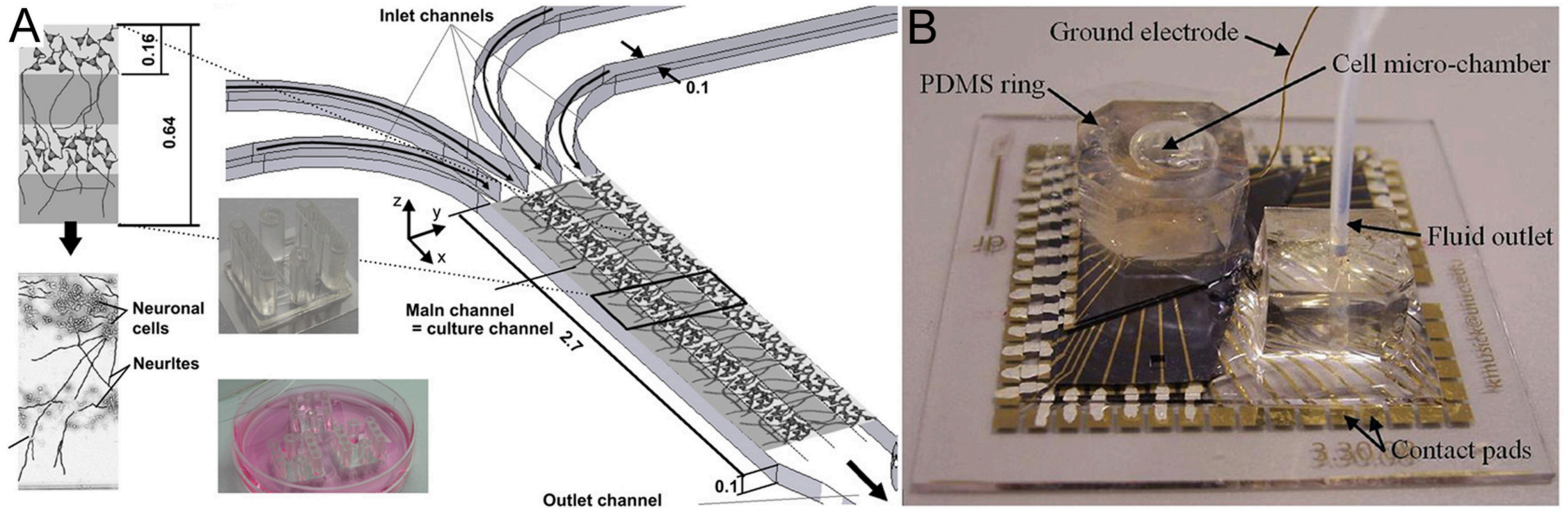
**(A)** Three-dimensional layers of the cerebral cortex, which were engineered using intercalating neuron-hydrogel layers. This drawing illustrates the layered structure of the 3D neural cell culture. The hydrogel or cell-loaded hydrogel flow through four inlet, main, and outlet channels. The two inserts show the final microfluidic device fabricated using polydimethylsiloxane (top insert) and three devices placed in a Petri dish for incubation during cell culture (bottom insert). Figure reprinted with permission, previously published in Biomaterials, Kunze et al. (2011a). **(B)** Three-dimensional microelectrode array for recording dissociated neuronal cultures. Figure reprinted with permission, previously published in Lab on a Chip, Musick et al. (2009).

These platform technology advances have enabled the production of various brain structures, including the cerebral cortical layers, which were engineered by intercalating neuron-hydrogel layers with plain hydrogel layers (Figure 3A; Kunze et al., 2011a). These engineered cortical layers exhibited different synaptic densities per layer as well as chemical gradients of growth factors (Cheng et al., 2007; Wong et al., 2008; Kunze et al., 2011c). In addition, neurospheroids, which are 3D non-hydrogel-based brain models, have also been developed (Choi et al., 2010). Choi et al. (2010) cultured cells from all six layers of the rat cortex at the bottom of concave microwells and investigated network formation inside of the neurospheroids. More detailed 3D brain models have also attempted to include the BBB using microfluidic approaches. The BBB is a 3D multicellular structure of the brain which regulates the passage of molecules from the blood to the brain and has profound implications for modeling disease responses to drugs (Vandenhaute et al., 2012; Banks, 2016). In BBB models, intersecting microfluidic channels are separated by a porous polycarbonate membrane upon which endothelial cells (vascular) and astrocytes (brain) are cultured on opposite sides, which essentially mimics the BBB (Griep et al., 2013). This membrane also allows for TEER measurements for barrier characterization (Odijk et al., 2015; van der Helm et al., 2016). Alternatively, a hollow fiber-like design (i.e., synthetic microvasculature, SyM-BBB) with enhanced visual capabilities has also been developed (Achyuta et al., 2013; Prabhakarpandian et al., 2013). These models have elucidated how drugs or toxins can breach the BBB and enter the brain microenvironment. Another brain model platform combines 3D cell cultures or samples with MEA systems, which allows for real-time electrical readouts of cells as well as the identification of electrical signatures associated with neurotoxicity (Pancrazio et al., 2003; Wölfer et al., 2006). Further advancement in this area has led to the development of 3D MEAs (Figure 3B) which convey 3D connectivity information (Köhling et al., 2005; Musick et al., 2009). Moreover, a system has been established that allows for 3D perfusion by using an active 3D microscaffold system with fluid perfusion for culturing *in vitro* neuronal networks (Rowe et al., 2007).

### Human Induced Pluripotent Stem Cell Technology

An alternative brain model that utilizes 3D culture involves the use of stem cell technology to engineer neural tissues which grow directly from neurospheres, yielding organoids (Lancaster et al., 2013). Although the aforementioned technologies allow for the development of brain-on-a-chip platforms, the cell sources used in such models must be carefully considered. Stem cell technology has been a giant-leap forward in the design of brain organoids; however, human induced pluripotent stem cells (hiPSCs) are an attractive alternative for on-chip brain modeling. HiPSCs have several advantages over immortalized neuronal cell lines or primary animal brain cells. HiPSCs can be obtained from human somatic cells (Takahashi et al., 2007) as an inexhaustible cellular resource. Moreover, hiPSCs can be cultured and differentiated into multiple brain cell types and are genetically matched with the patient (Dolmetsch and Geschwind, 2011). HiPSCs differentiated into neural lineages allow for neurotoxicological or neurodevelopmental assays as well as the analysis of mature human neuronal networks by exploiting self-organization during neural differentiation. Kilic et al. (2016) demonstrated the feasibility of differentiating pluripotent human cells (NTERA2) into neuronal clusters containing astrocytes, which interfaced with a layer of human brain microvascular endothelial cells that had BBB characteristics. This 3D multicellular on-chip environment enhanced chemotactic cue-induced human neural progenitor migration (i.e., CXCL12 expressed during embryonic brain development and in pathological CNS tissues). A promising development in iPSC technology is the use of patient-derived iPSCs containing single mutations that lead to disease (e.g., familial dysautonomia; Lee et al., 2009). In such patients, the IKBKAP encoding gene contains a point mutation that is directly correlated with the loss of autonomic and sensory neurons. Through healthy vs. diseased hiPSC screening, the collection of patient-derived iPSCs can allow for diagnosis and *in vitro* drug treatment prior to patient treatment, which could lead to more personal and efficient diagnoses and drug treatments (Park et al., 2008; Dolmetsch and Geschwind, 2011).

### Disease Models

Neurodegenerative diseases and disorders, such as Parkinson's disease (PD) and Alzheimer's disease (AD), lead to the destruction or degradation of synaptic connections, whereas neurological diseases, such as epilepsy, are thought to be related to dysfunctional network responses. Although epilepsy-on-a-chip has not been established yet, brain-on-a-chip technology has been applied to PD and AD modeling. In the following sections, we discuss the main technical features of the brain-on-a-chip platforms utilized for these disease models.

#### Alzheimer's Disease-On-A-Chip

**Figure 4:**
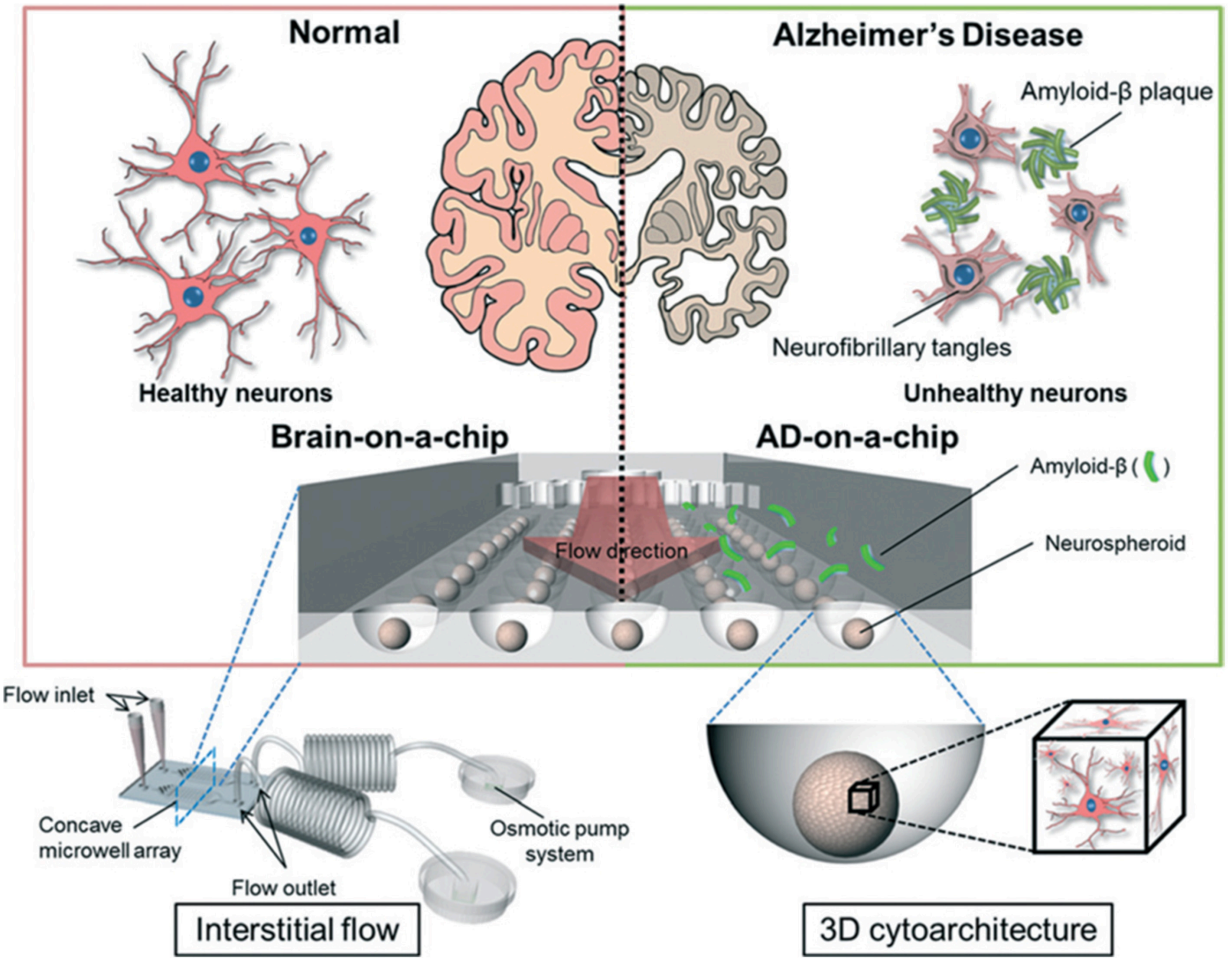
Schematic diagram of a 3D Alzheimer's disease brain-on-a-chip with an interstitial level of flow. The chip contains a concave microwell array for the formation of homogeneous neurospheroids of a uniform size with 3D cytoarchitecture. The osmotic micropump system is connected to the outlet to provide a continuous flow of medium at the level of interstitial flow. By providing 3D cytoarchitecture and interstitial flow, this chip approximates the microenvironment of normal and Alzheimer's disease brains, which facilitates the investigation of amyloid-β effects on 3D neural tissue. Figure reprinted with permission, previously published in Lab on a Chip, Park et al. (2015).

In AD, synaptic dysfunction is usually related to malfunctions of proteins, such as tau and amyloid beta (amyloid-β) (Pascoal et al., 2016). Therefore, some AD models have focused on modulating these proteins with respect to their influence on synapse formation and glial cell communication (Hai et al., 2010). Three-dimensional neuronal tissue models, including the aforementioned networked neurospheres (Choi et al., 2010), have also been used for AD studies investigating amyloid-β protein expression and network formation (Choi et al., 2013). Platforms and systems offering real-time analyses of neuronal activity, co-culturing, and chemotaxis gradients have been used to model AD. Previously, microfluidics were successfully used to demonstrate the role of amyloid-β in neuronal connections (Deleglise et al., 2014) and glial cells (Cho et al., 2013). Using these models, these studies showed that amyloid-β accumulation in cortical neurons led to the occurrence of synaptic anomalies at the level of neurotransmitter signaling pathways, which represented the onset of AD. Tau protein hyperphosphorylation is a hallmark trait of AD (Pascoal et al., 2016), and studies combining microfluidics and co-cultures demonstrated that different tau phosphorylation states could be modeled within interconnected microfluidic neuronal cell compartments (Kunze et al., 2011b; Cho et al., 2013). In another AD model study, microfluidic devices were used to demonstrate neuron-to-neuron wild-type tau protein transfer through trans-synaptic mechanisms (Dujardin et al., 2014). More recently, an 3D on-chip AD model was proposed (Park et al., 2015; Figure 4). The use of microfluidic technologies allowed for culture media perfusion to assess perfused amyloid-β effects on network formation (Park et al., 2015). This chip contained concave microwells for the formation of homogeneous 3D neurospheroids of a uniform size. Its osmotic micropump system was connected to the outlet to provide a continuous flow of medium. By providing 3D cytoarchitecture and interstitial flow, this chip approximated the microenvironment of normal and AD brains, which facilitated the investigation of amyloid-β effects on 3D neural tissue. On the microfluidic chip, normal (i.e., healthy) neurospheroids were cultured under dynamic conditions with a flow of normal medium containing oxygen and nutrients for 10 d. AD neurospheroids were cultured on the microfluidic chip under dynamic conditions with a flow of normal medium containing oxygen and nutrients for 7 d. Following this, the AD neurospheroids were incubated with a medium containing 5 μM of synthetic amyloid-β for 3 d. Compared with the normal model, the AD model had decreased cell viability and increased neural destruction and synaptic dysfunction, which are pathophysiological features of AD *in vivo*.

#### Parkinson's Disease-On-A-Chip

An on-chip PD model that allowed for monitoring of mitochondrial transport on single dopaminergic axons was also proposed (Lu et al., 2012). This device consisted of two open chambers connected via microchannels in which axon growth was monitored and labeled mitochondria were visualized. The device promoted oriented axon growth into a separate axonal compartment for analysis. Moreover, this device improved upon the culture of more sensitive neurons (i.e., primary midbrain dopaminergic neurons). Although this work was limited to mitochondrial transport, vesicular transport, and microtubule fragmentation, which also contribute to dopaminergic fiber loss, could also be analyzed with this device. This is an important development because such studies are difficult to perform using traditional cell culture approaches and PD brain lesions are always associated with dopaminergic fiber loss. To date, this device does not support co-culture or 3D cell cultures but instead uses microfluidics to align axons. This approach highlights the axonal degeneration mechanisms potentially underlying PD pathophysiology as well as those underlying other major neurodegenerative diseases.

Another potential on-chip PD model using 3D phase-guided microfluidic cell culture bioreactors was recently developed as a personalized biomedical approach to PD (Moreno et al., 2015). These authors differentiated human neuroepithelial stem cells into dopaminergic neurons in microfluidic cell culture bioreactors and suggested that this platform could be used to study substantia nigra dopaminergic neuron degeneration, a hallmark of PD.

#### Epilepsy

Epilepsy is characterized by excessive synchronized electrical activity within the brain. MEAs in combination with brain slices have been the best technology used so far to monitor, study, and detect epileptic activity *in vitro*. This technology consists of rapid high-throughput static platforms used for drug discovery and toxicology studies. Recently, an *in vitro* model of spontaneous epilepsy was proposed in which cells cultured from transgenic mice expressing β2-V287L were used with MEA technology to assess the role of Bβ2-V287L in synaptic formation (Gullo et al., 2014). With this β2-V287L model, the authors showed that it is possible to produce murine models with human channelopathy *in vitro*. In addition, MEAs coupled with microfluidics have been proposed to monitor neuronal network activity under different conditions and exposures (Morin et al., 2006; Ravula et al., 2006). These brain slice-based models can be used as chronic models of spontaneous hyperexcitability (i.e., epileptiform) activity, which does not require pre-treatment with pharmacological agents to trigger seizures. However, these models have questionable clinical relevance. Given the recent advances combining brain-on-a-chip and iPSC technologies, epilepsy-on-a-chip may become a reality. Genetic factors play an important etiological role in epilepsy development. Harvesting hiPSCs from patients with Dravet syndrome, in which a single SCN1A gene mutation causes epilepsy (Selmer et al., 2009), holds great promise for enhancing treatment options and improving quality of life.

## Future Directions of Clinically Validated On-Chip Brain Models

Although tremendous progress has been made with brain-on-a-chip technology and its applications, challenges remain with respect to the translational and clinical value of such systems. To recreate the critical features of the *in vivo* human brain microenvironment, several factors must be addressed. First, advanced cell composition reflecting the type, ratio, and 3D architecture of cells within brain tissue must be achieved by incorporating stem cell technologies (Lancaster et al., 2013; Berger et al., 2018). Another important aspect of validating brain-on-a-chip models is to identify the functional readouts of the healthy or pathological states of these systems. As previously discussed, MEA-based electrophysiological measurements should be appropriate for 3D microphysiological cell culture systems. Alternatively, advanced neuroprobe microtechnologies (Xie et al., 2015) are useful for assessing brain function; however, these tools remain to be implemented in brain-on-a-chip models. Progress in neuroprobe technology has been reviewed elsewhere (Seymour et al., 2017).

Despite the current limitations, most modern microscale platforms have already identified known pathological mechanisms and pathways; however, they have yet to contribute to novel therapeutic solutions. Thus, the clinical relevance of brain-on-a-chip technology remains limited; however, the pharmaceutical industry has started to examine these new technologies for drug discovery and testing. Nonetheless, these systems show great promise in more closely representing the diseased human tissue microenvironment *in vitro* compared with standard tissue culture tests. Resolving the aforementioned critical features as well as mimicking blood flow and the BBB, or neurovascular unit, will be important steps toward advancing clinically relevant brain-on-a-chip models. The next sections will describe an idealized schematic of a brain-on-a-chip concept, which details its desired physiological features. Moreover, we address novel assay development routes that exploit 3D engineered tissue architecture and provide a market outlook.

### Nano- and Microfabrication Challenges With Brain-On-a-Chip Technology

Essentially, a brain-on-a-chip is a miniaturized dish-type construct placed on a microscope slide, which hosts neuronal tissue supported by a medium replenishment unit and integrated microfluidics. To produce such a device, the appropriate cells must first be selected. This step is critical because brain tissue consists of many different neuronal subtypes (Brodal, 2010). Moreover, a plethora of supporting glial cell types, including microglia, astrocytes and oligodendrocytes, are also required to design advanced brain-on-a-chip models. To further complicate matters, cellular network bio-architecture significantly varies throughout different brain regions, forming complex structures and circuitries. Importantly, different cell types appear in precise ratios in different brain regions, where, for example, glial cells influence apoptosis and repair mechanisms and can accumulate following brain trauma (Eskes et al., 2003; Giordano et al., 2009; Kuegler et al., 2012). Glial cells also determine the overall reaction of tissue to injury by phagocytosing dying neurons and neurite debris (Hirt and Leist, 2003). Therefore, brain injury models need to reflect these altered conditions instead of only modeling healthy brain function. In addition, several metabolic pathways in the brain involve different cell types. For example, astrocytes take up glutamate, transform it to glutamine, and provide it to neurons. Astrocytes also provide neurons with specific energy substrates or essential thiols. Therefore, co-culturing of different cells must be performed in diseased or healthy brain-on-a-chip models.

**Figure 5:**
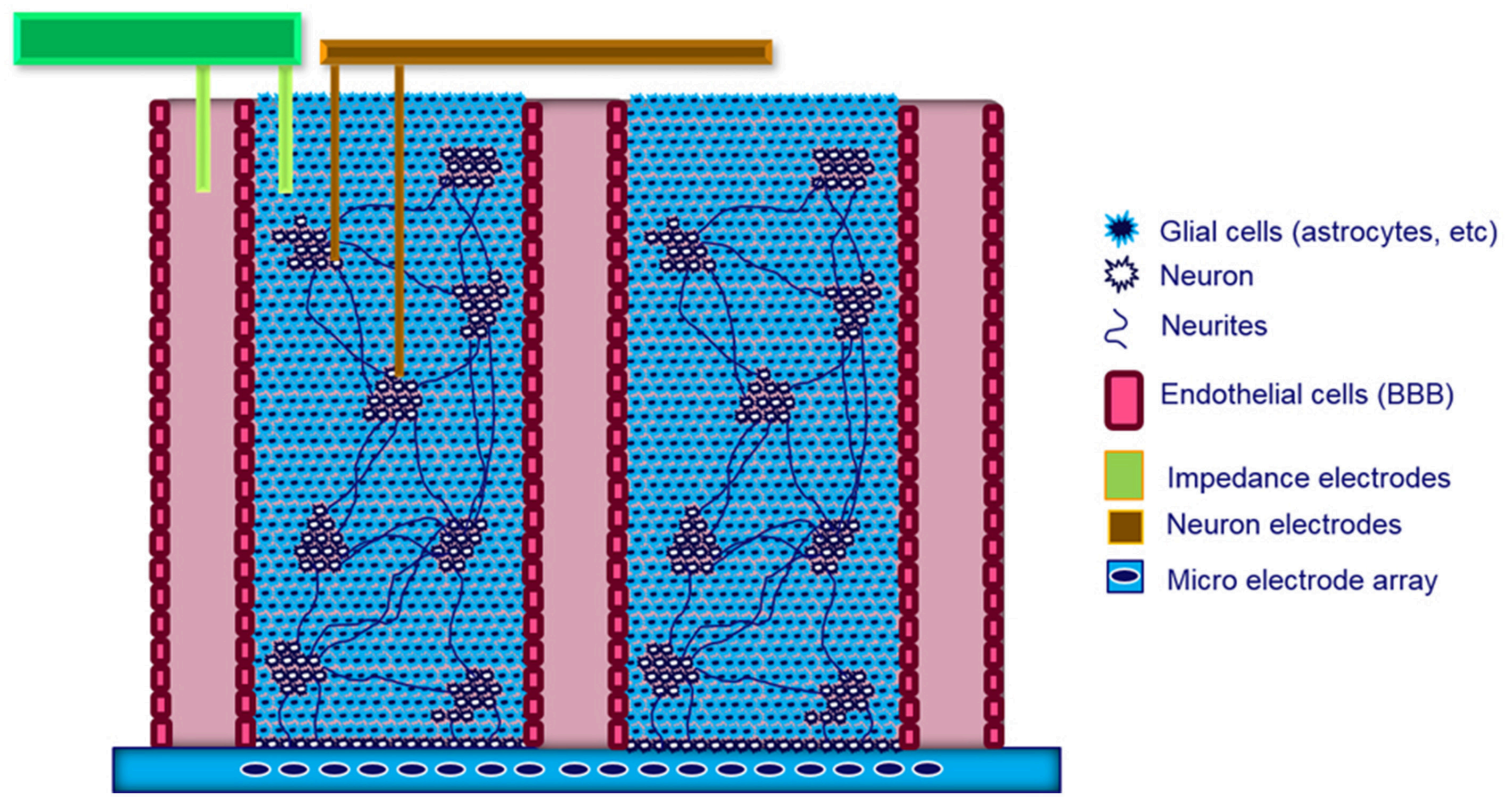
Schematic side view of the ideal brain model. This model has a cell composition of 10% neurons and 90% glial cells. The 3D architecture, which includes the blood-brain barrier (BBB), is interfaced with a microelectrode array (MEA). Additional output measurements can be made using external electrodes.

For the brain model to be functional, all cell types must be present and supported by the engineered construct (Figure 5). This may be achieved by combining engineered microsystems with hiPSC technology. HiPSC-derived neuronal cell cultures are beneficial in this respect because they can contribute to a specific tissue architecture based on their level of differentiation. Differentiation can be induced within particular compartmentalized microenvironments specifically designed within the microphysiological cell culture system to fulfill the requirements of a given application. Preliminary successes in this area have been recently demonstrated, amongst others, by Fleming and his team who utilized the Mimetas platform (Moreno et al., 2015). However, in that system, the 3D space was limited to a few hundred micrometers and there was no electrical readout.

Unlike organoid culture in flasks (Lancaster et al., 2013), brain-on-a-chip models have only been developed with a maximum of two or three cell types, which is not sufficient to achieve fully functional brain tissue. In addition to cell type and complexity issues, the host environment must allow for sufficient medium exchange with different cell types that may require dedicated media and growth factors. For example, neuron and astrocyte culture media differ during differentiation, which often requires a flow barrier that prevents direct co-culture and connection between the two cell types. Future micro- and nanofluidic systems of the brain should specifically address these technical challenges to obtain representative physiological behavior within brain-on-a-chip models and, thus, advance their clinical relevance.

### 3D Engineered Tissue Architecture-Based Assays

In brain-on-a-chip models, the cell construct architecture should resemble the spatial distribution of cells within tissue, allowing for 3D culture instead of the conventional 2D conformation produced by many high-throughput cell culture screening platforms (Frimat et al., 2010; Hardelauf et al., 2011). A 3D conformation will affect the neuronal network (i.e., connectivity) and consequently, the signals sent between cells (Frega et al., 2014). This, in turn, would lead to changes in metabolism and neuron activity. Three-dimensional models often result in improved structure, which is demonstrated by cellular outgrowths (i.e., neuronal processes) and enhanced cell-to-cell connectivity. Moreover, 3D cultures often show enhanced survival and richer neuronal differentiation compared with traditional monolayer cultures (Peretz et al., 2007). Local secretion and paracrine signaling of neurotrophic factors (e.g., nerve growth factor and brain-derived neurotrophic factor) together with other intercellular communication events are also desired features that are important for recreating brain function within a model (Maurel and Salzer, 2000; Michailov et al., 2004). Designing 3D *in vitro* models to enable this type of bio-architecture is therefore an important consideration for brain-on-a-chip development. Functional synapses have been observed earlier in 3D neuronal cultures in collagen hydrogel compared with 2D models (O'Shaughnessy et al., 2003). Furthermore, stem cell-derived neurons cultured in 3D showed increased differentiation compared with 2D cultures (Paavilainen et al., 2018). Morphology at the single-cell level is also different in 3D, where cells adopt a round shape compared with cells that spread on 2D surfaces, which influences cells at the genetic level (Li et al., 2007). For example, genetic and morphological microarray analysis of neurons growing in gels (3D) compared with those cultured on standard tissue culture plates (2D) demonstrated that cells cultured in 3D exhibited differential expression of 1,766 genes, including those relevant to cytoskeleton, extracellular matrix, and neurite outgrowth (e.g., filamin A, actinin 1a1, capping protein a2, fibronectin 1, and midkine; Li et al., 2007). In addition, when 3D cultured in gels (e.g., collagen, Matrigel, or Puramatrix), neurons and other cell types adopted a more *in vivo*-like phenotype, including more branched and thicker neuronal processes compared with those cultured in 2D (Ylä-Outinen et al., 2014). The *in vivo* structural features of brain tissue bio-architecture support network formation and regionally compartmentalize the brain to connect different regions and allow for cognitive processing (Honegger et al., 2016). Therefore, the architecture of the brain is not random, and during development, 3D structural modification occurs inside the brain. Moreover, brain architecture is highly heterogeneous and does not have repeating units or exhibit clear structural patterns. Therefore, to generate a brain model *in vitro*, different brain regions (e.g., the medulla, pons, hypothalamus, thalamus, cerebellum, optic tectum, pallium, hippocampus, basal ganglia, and olfactory bulb) should be modeled separately at first to develop minimal brain model concepts. These regionally specified brain-on-a-chip models should be arranged and interconnected in a way resembling a real brain to permit cross-talk between regions, which could potentially be facilitated by an electronic link (Panuccio et al., 2016).

Considering the above issues, 3D assays must be developed. Nano- and microfabrication technologies can help to reach this goal with nano-patterning (e.g., nanoimprint and microcontact printing; Martínez et al., 2012), additive manufacturing routes (e.g., microprinting; Marga et al., 2012; Ye et al., 2018), and hydrogel templating (Matsusaki et al., 2007) approaches. These techniques can subsequently assist in deriving the desired 3D assemblies for augmenting cell-to-cell and cell-to-substrate interactions. Our research has shown that nanogroove substrates produced by replica molding from a nanoimprint lithography template can impact culture organization in 3D (Frimat et al., 2015). Moreover, self-assembly of cell-laden hydrogel beads, produced by flow-focusing microfluidics, can yield novel 3D cell culture arrangements, which can facilitate assay development due to the regular ordering of neurons in culture (Bastiaens, 2019).

### Potential for Micro-Nanofluidics Systems of the Brain

BBB devices are often considered to be brain-on-a-chip models; however, they are not. BBB-type devices monitor what enters and exits the brain and, although BBB function can be mimicked *in vitro* (Griep et al., 2013; van der Helm et al., 2016), it is usually not coupled with a brain model. Given that the BBB is essential for the brain and its functions, it should be included in brain models as a building block for the compartmentalization of different brain regions. A stand-alone human BBB-on-a-chip, or neurovascular unit, was recently developed and serves as a first step toward improving drug development (Heidari and Taylor, 2018). The structural components of BBB blood vessels can serve as feeding channels for more advanced culture models including immune system cells. Optimizing the 3D *in vitro* brain microenvironment, which should include extracellular matrix (ECM) modeling to recreate *in vivo* brain physiology better than that of the current state-of-the art, can be achieved by including micro- and nanofluidic technology in addition to the appropriate cell types. Continuous medium supply and realistic 3D conformation are prerequisites for differentiating multiple cell types within a model and sustaining 3D cultures for prolonged time periods. A vascular ECM-type scaffold design may allow for highly specific cellular niches to be established by providing continuous or local access to growth factors, correct cell matrix adhesion (i.e., polarity), nutrition, cell-to-cell junction formation, and immune system (i.e., T-cell) modeling (Kipnis and Schwartz, 2005). With exquisite mimicking facilitated by micro- and nanofluidics, such 3D reconstructions could eventually provide an economical platform to model stem cell niches, which would enable specific cell compositions and signaling molecule gradients. These developments would provide a highly defined environment (e.g., enriched or deprived of certain signaling molecules) for the development and functioning of cultured brain tissues, which could overcome current difficulties in securing scarcely available brain tissue slices. During neuronal development, signaling molecule gradients are particularly important for cell migration and differentiation and, thus, overall brain structure patterning. From a clinical perspective, 3D cell culture systems have obvious advantages over 2D systems when modeling for a specific research question is the major objective (Fuchs et al., 2004; Morrison and Spradling, 2008). However, if a 3D cell culture model is not engineered properly or its matrix (typically hydrogels) is not porous enough, there can be limited diffusion of chemicals and nutrients and signals can be lost. In the brain, a cell is never more than 200 μm away from a blood vessel. This feature should be replicated in brain-on-a-chip applications to create marketable products and services with highly robust experimental designs.

## Conclusion

Although the union of micro- and nanotechnologies with neuroscience has been demonstrated by a number of microfluidic devices, the lack of a deeper understanding of the brain still hampers the selection of appropriate design criteria. To overcome these design bottlenecks, it is important to understand the clinical and pharmaceutical requirements for organ-on-a-chip technologies, which can revolutionize biology and personal medicine. Conventional neuronal cell culture methods have limited relevance for brain disease modeling. The combination of microfluidic, MEA, and iPSC technologies has had a significant impact on the development of brain-on-a-chip technology. We have discussed microfluidic technologies that emulate the BBB, or neurovascular unit, and establish nutrient replenishment routes with distinguishable growth factor gradients as well as compartmentalized culture systems. Furthermore, current brain-on-a-chip models are limited to immunostaining or 2D MEA readouts. Therefore, 3D culture could benefit from the integration of advanced neuroprobe technology and 3D cultures. In addition, stem cell technology must improve the differentiation methods for multicellular tissue constructs. Overall, on-chip integration methods have already enabled advances in AD, PD, and epilepsy modeling. In AD modeling, microfluidic approaches have provided drug screening advantages. Moreover, cell soma and axon compartmentalization and visualization have been greatly improved in PD models. In epilepsy, electrode integration and genetic manipulation will allow for new insights into specific disease mechanisms. Integrating different features into one compact brain-on-a-chip model is a major technical challenge in this field. For actual brain models, the integration of microfluidic neurovascular units and electrophysiological readouts in 3D multicellular tissue constructs will be important steps forward.

## Author Contributions

J-PF and RL: conceptualization and design of the review, preparation of manuscript, and final approval of the version to be published. RL: acquired the funding.

### Conflict of Interest Statement

The authors declare that the research was conducted in the absence of any commercial or financial relationships that could be construed as a potential conflict of interest.
